# A fully automated crystallization apparatus for small protein quantities

**DOI:** 10.1107/S2053230X20015514

**Published:** 2021-01-01

**Authors:** Ryuichi Kato, Masahiko Hiraki, Yusuke Yamada, Mikio Tanabe, Toshiya Senda

**Affiliations:** a Structural Biology Research Center, Institute of Materials Structure Science, High Energy Accelerator Research Organization (KEK), Oho 1-1, Tsukuba, Ibaraki 305-0801, Japan; b Institute of Particle and Nuclear Studies, High Energy Accelerator Research Organization (KEK), Oho 1-1, Tsukuba, Ibaraki 305-0801, Japan

**Keywords:** crystallization, automation, high-throughput, membrane proteins

## Abstract

In 2003, a fully automated protein crystallization and monitoring system (PXS) was developed to support the structural genomics projects that were initiated in the early 2000s. In PXS, crystallization plates were automatically set up using the vapor-diffusion method, transferred to incubators and automatically observed according to a pre-set schedule. The captured images of each crystallization drop could be monitored through the internet using a web browser. While the screening throughput of PXS was very high, the demands of users have gradually changed over the ensuing years. To study difficult proteins, it has become important to screen crystallization conditions using small amounts of proteins. Moreover, membrane proteins have become one of the main targets for X-ray crystallography. Therefore, to meet the evolving demands of users, PXS was upgraded to PXS2. In PXS2, the minimum volume of the dispenser is reduced to 0.1 µl to minimize the amount of sample, and the resolution of the captured images is increased to five million pixels in order to observe small crystallization drops in detail. In addition to the 20°C incubators, a 4°C incubator was installed in PXS2 because crystallization results may vary with temperature. To support membrane-protein crystallization, PXS2 includes a procedure for the bicelle method. In addition, the system supports a lipidic cubic phase (LCP) method that uses a film sandwich plate and that was specifically designed for PXS2. These improvements expand the applicability of PXS2, reducing the bottleneck of X-ray protein crystallography.

## Abstract 2

An improved, fully automated protein crystallization and monitoring system, PXS2, can perform crystallization using smaller protein samples than the previous version. In addition, PXS2 can be used for membrane-protein crystallization using the bicelle and LCP methods.

## 1. Introduction   

Three-dimensional structural information on proteins and other biological macromolecules is important not only to elucidate the molecular mechanisms of biochemical processes in living organisms, but also to accelerate drug-discovery processes. Recent advances in the single-particle analysis method using cryo-electron microscopy (cryo-EM) have allowed us to determine protein structures at subatomic resolution with small sample volumes without crystallization (Liao *et al.*, 2013 ▸; Cheng, 2018 ▸). However, it is still challenging to determine the structures of small proteins with a molecular mass of less than 40 kDa by single-particle analysis using cryo-EM. Macromolecular X-ray crystallography (MX) has been widely used to determine protein and protein–ligand complex structures. Especially in pharmaceutical science, high-throughput drug screening using MX is still a critical method. Once a very high-quality crystal has been obtained, the crystal structure can be determined without laborious efforts owing to recent improvements in synchrotron beamlines, including the development of faster detectors, and improved structure-analysis software. Crystallization screening requires a large amount of sample and sometimes takes a long time, and is therefore the most critical step in MX.

At the same time, difficulty in obtaining a high-quality crystal remains a major bottleneck in MX. Over the last two decades, several academic and industrial groups interested in MX have developed automation for crystallization screening (Hiraki *et al.*, 2006 ▸; Sugahara *et al.*, 2008 ▸; Gorrec & Löwe, 2018 ▸; Weber *et al.*, 2019 ▸). These machines were designed for high-throughput crystallization screening, mainly in structural genomics projects. Typically, the systems are composed of two functional parts: automatic dispensers to set up the crystallization drops and microscopes with cameras to observe the drops automatically. Developments are limited at the moment, and only a limited number of independent dispensers and microscopes that can function as standalone automated machines are commercially available. Since fully automated systems including all of the steps from crystallization-plate setup to drop observation are necessarily several modules controlled by multiple computer programs, their development is complicated. On the other hand, standalone automated machines with limited functions are easy to use and maintain. However, when aiming at fully automated MX for high-throughput analysis at synchrotron facilities, the development of more comprehensive systems is still critically important.

In 2003, we developed a fully automated protein crystallization and monitoring system (PXS) to achieve high-throughput crystallization screening for a structural genomics project (Hiraki *et al.*, 2006 ▸). PXS made a great contribution to initial crystallization screening for 14 years and set up 9465 plates for 70 users (907 640 drops). PXS users have requested various improvements to the PXS system in order to pursue difficult structural biology targets such as membrane proteins. In addition, significant advances have been made in automation in the MX field, especially at synchrotron beamlines, such as crystal mounting on the goniometer, crystal centering, data collection and data processing. Some synchrotron facilities are moving to integrate these automated experimental processes in order to achieve fully automated crystallography from crystallization to structure determination. Automated crystallization screening is considered to be one of the key components in this process. In addition, this type of integration requires the integration of experimental databases. We therefore developed a new database system for PXS2 and are now working to integrate this database with PReMo (PF Remote Monitoring System; Yamada *et al.*, 2013 ▸), which includes a database for diffraction data collection on the Photon Factory (PF) synchrotron beamlines. Here, we describe the upgrade of PXS to PXS2, which has several new functions as well as improved performance for more efficient and more versatile MX.

## 2. Results   

### 2.1. Presentation of PXS and its upgrade plan   

**Figure 1:**
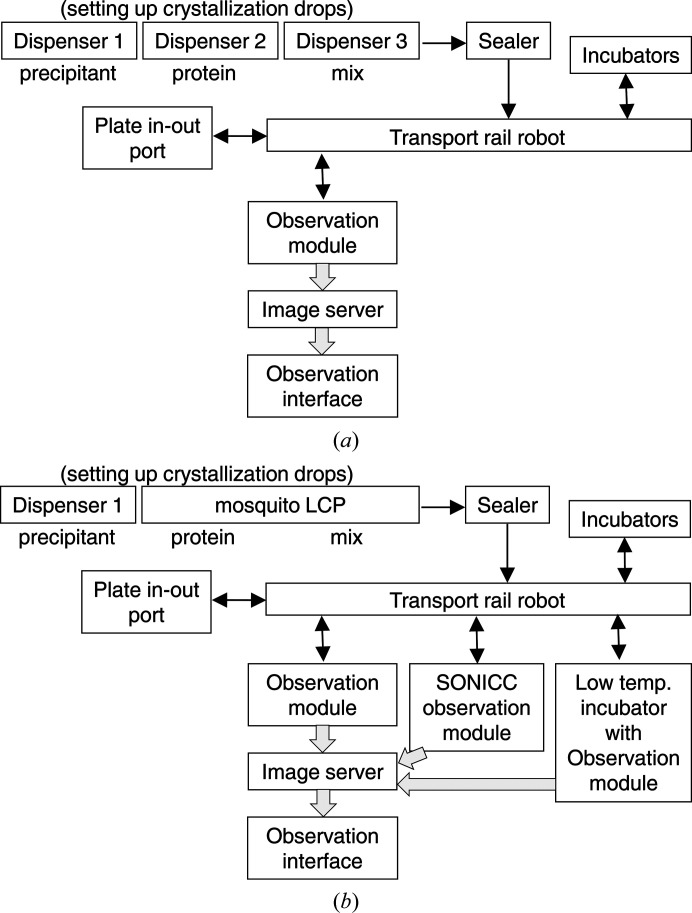
Schematic diagram of the fully automated protein crystallization and monitoring system. Solid arrows indicate the motion of the crystallization plates, and gray boxed arrows indicate the flow of the captured image data. (*a*) In the original PXS, three dispensers work to dispense the crystallization (precipitant) and protein solutions and to mix them. Crystallization plates are transported among the dispensers by a shuttle transport device. (*b*) In the improved system, PXS2, two dispensers and an articulated robot work to complete the crystallization drops. A SONICC observation module and a low-temperature incubator with an observation module are newly installed.

The original PXS had a modular design (Hiraki *et al.*, 2006 ▸): the system comprised six standalone modules, each of which could function independently and be replaced separately. PXS consisted of the following modules: (i) dispensers for crystallization (precipitant) and protein solutions, (ii) a plate sealer, (iii) a plate-transport rail robot, (iv) plate incubators, (v) an observation module and (vi) an image-storage server (Fig. 1 ▸ *a*). Users could operate PXS using a single control software; PXS automatically performed all steps from crystallization setup to crystallization-drop observation. The images were acquired automatically according to a user-input schedule, and were accessible via the internet using a web browser. At the time of its development, PXS was the fastest system in the world for setting up crystallization drops; it required only 36 s to set up crystallization in a 96-well crystallization plate.

After 14 years of operation, we decided to upgrade PXS in order to meet the latest requirements of MX. Considering the advancements in the MX field, four major updates were needed in PXS: a reduction of the dispensing volume to minimize the amount of sample required for difficult proteins, high-resolution imaging to detect small crystals, the addition of an incubator at 4°C to increase the success rate of crystallization, and crystallization screening of membrane proteins. Based on these requirements, we upgraded PXS to PXS2. In this upgrade, we prioritized reduction of the dispensed sample volumes over increasing the dispensing speed. In addition, we decided to continue using the KEK crystallization plate in PXS2, because the plate-handling robots in PXS2 and the *in situ* data-collection system at the PF beamlines (Yamada *et al.*, 2016 ▸) have been adapted for use with the KEK plate. The KEK plate is an SBS-formatted 96-well crystallization plate designed for PXS (Hiraki *et al.*, 2006 ▸). The crystallization (precipitant) solutions used in PXS2 must be provided in SBS-formatted 96-deep-well plates. In this way, PXS2 will be able to use either a commercially available or an in-house crystallization screening solution. In addition, we can use the original crystallization solution for optimization if the solution is prepared in a 96-deep-well plate. The deep-well plates and KEK crystallization plates are labeled with barcodes for automated management of the crystallization conditions. A diagram of PXS2 is shown in Fig. 1 ▸(*b*) and its specifications are summarized in Table 1 ▸.

### 2.2. Improvements   

#### 2.2.1. Crystallization-plate setup module   

**Figure 2:**
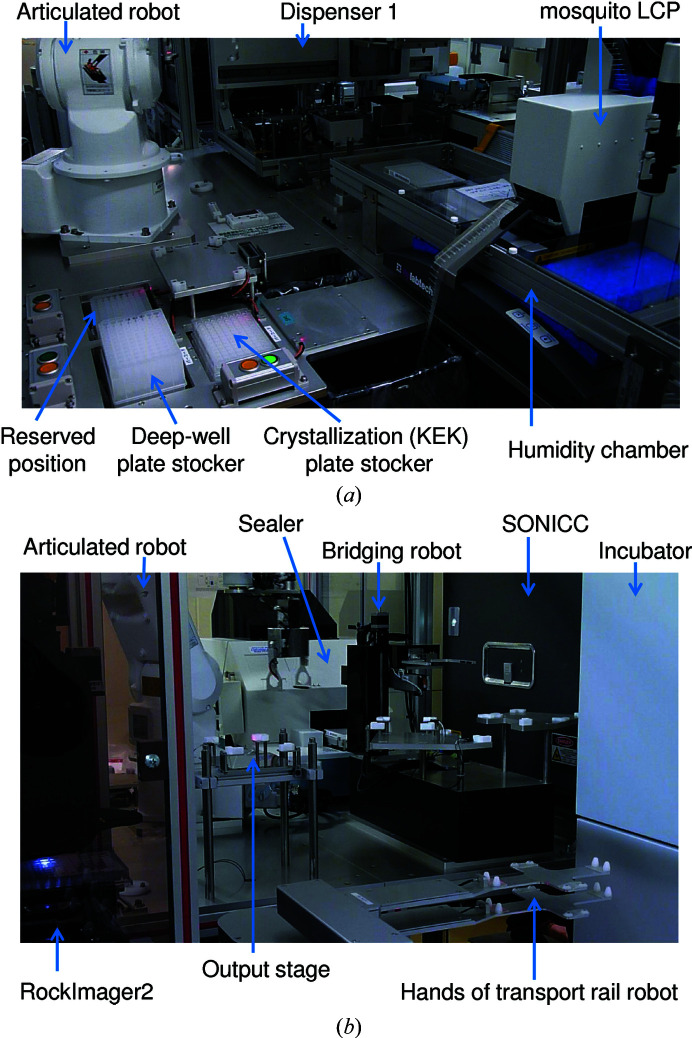
Photographs of PXS2, mainly showing the mechanism for setting up the crystallization plates. (*a*) A view showing dispenser 1 and the mosquito LCP. Plate stockers and the articulated robot are also shown. (*b*) A view from the opposite side showing the sealer. The articulated robot, the bridging robot, imagers (RockImager2 and SONICC), one of the incubators and the hands of the transport rail robot are also shown.

To set up the crystallization drops, three dispensing modes are needed: a mode for dispensing crystallization (precipitant) solution to the KEK plate, a mode for dispensing a protein sample to the KEK plate, and a mode for mixing the precipitant and protein solutions. For the first mode, we overhauled dispenser 1 of PXS, which used disposable tips with 96 dispensing heads, and installed it on PXS2 (Figs. 1 ▸ and 2 ▸ *a*). We evaluated the coefficient of variation (CV) values using water, 30% 2-propanol and 30% PEG 8000, and found that they were less than 3% when dispensing samples of 100 µl. In the second and third dispensing modes, PXS2 requires that small volumes be dispensed in order to reduce the sample volume. We therefore installed a mosquito LCP (SPT Labtech, UK) with a humidity chamber to prevent the evaporation of small drops (Fig. 2 ▸ *a*). The protein sample is provided in an SBS-formatted 384-well plate which is supplied to the mosquito LCP. We can set up crystallization drops with microseeds by using the continuous aspiration function of the mosquito LCP, which helps to optimize the crystallization conditions.

We installed an articulated robot (MOTOMAN-MH3F; Yaskawa Electric Corporation, Japan) with a hand that can grab and transport the KEK plate and a 96-deep-well plate between plate stockers and dispensers (Fig. 2 ▸ *a*). After completion of the crystallization-drop setup, the articulated robot brings the KEK plate to the sealer and then to the output stage (Fig. 2 ▸ *b*). PXS2 can set up a maximum of eight crystallization plates at once. A transport rail robot (RR757L15-K2; Rorze Corporation, Japan) promptly transfers the plate from the output stage to the observation module or an incubator.

The film used to seal the KEK plate was changed from a glue film (Hiraki *et al.*, 2006 ▸) to 3M CNNT-150 film, which is transparent with nonpolarization and low vapor permeability. The film has no adhesive on its own surface, but the adhesive components are extruded to bond the film to the plate when pressure is applied. We installed a sealer, PS-2002 (Micronics Inc., Japan), which is dedicated to the 3M CNNT-150 film supplied as a 150 m long roll (Fig. 2 ▸ *b*). The movements of the dispensers, articulated robot and sealer are controlled by our software in a coordinated manner.

#### 2.2.2. Observation module   

The observation module was upgraded from a VGA resolution (0.3 million pixels) color CCD camera with a fixed-angle polarizer to a higher resolution (five million pixels) observation module that is specialized for protein crystallography (RockImager2; Formulatrix, Massachusetts, USA; Fig. 2 ▸ *b*). In addition to the RockImager2, we installed a benchtop SONICC (Formulatrix), which can image crystals by second-harmonic generation (SHG) and ultraviolet two-photon excited fluorescence (UV-TPEF). SHG is a technique that can detect small crystals or crystals in a turbid drop (Wampler *et al.*, 2008 ▸). UV-TPEF is used to detect autofluorescence from protein crystals when they contain aromatic amino-acid residues such as tryptophan and tyrosine (Madden *et al.*, 2011 ▸). A bridging robot connecting the SONICC and the transport rail robot was developed and installed (Fig. 2 ▸ *b*).

**Figure 3:**
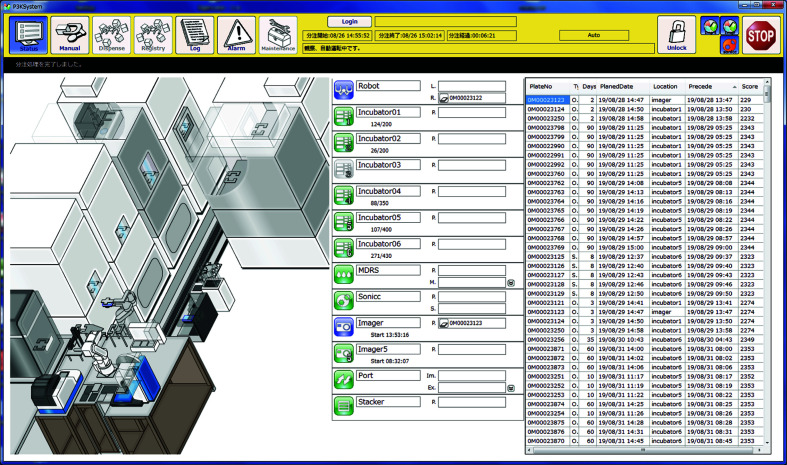
Graphical user interface (GUI) of the control software of PXS2. The seven icons at the top left of the screen are links to the respective screens with different functions. In this screen, the left part shows the current status of PXS2 by means of a simple animation. The center portion of the screen shows the status of each component by color: green, blue, gray and red represent ready, working, out of service and trouble, respectively. The table on the right shows the observation schedule.

**Figure 4:**
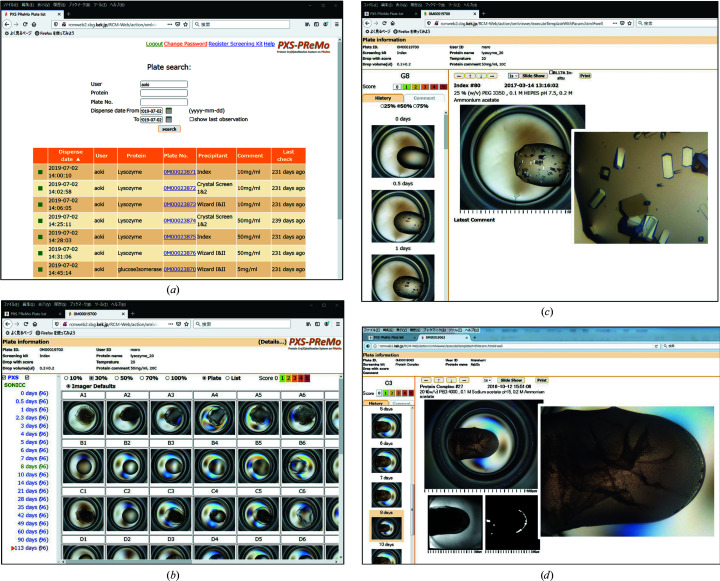
Operation screens of PXS-PReMo, which can be accessed via the internet using a web browser. (*a*) Users can search their crystallization plates by sample name, plate ID or dispensed date from the plate list after logging in. (*b*) A window opens in a new tab when the user selects one plate. All 96-well images at the latest observation date are displayed. (*c*) Detailed information on each drop can be displayed when one well is selected in the 96-well images in (*b*). In the left column, chronological development is shown. A full-size image at each observation time is displayed in the center of the screen. A new window of the picture appears by clicking the full-size image, and the picture in the window can be expanded by wheeling the mouse on the picture. (*d*) Viewing screen of the SONICC observation. Three images obtained by SONICC, *i.e.* the usual optical, UV-TPEF and SHG images, are shown as tiles in the center of the screen. Each image can be expanded by clicking and wheeling the mouse.

These observation modules are operated by external software which we developed and synchronized with the transport of the plate (Fig. 3 ▸). All data, including the acquired images and crystallization conditions, are managed by our own database developed with XML-based middleware: the RCM system (Quatre-i Science Inc., Japan). The data-handling architecture was completely changed from the original PXS to include a function for communication with other databases, but the user interface was kept almost the same to preserve usability (Fig. 4 ▸). The whole system, including the database and the GUI, is called PXS-PReMo, which was named after PReMo, a database system for diffraction data collection at PF (Yamada *et al.*, 2013 ▸).

#### 2.2.3. Incubators   

PXS2 has five incubators at 20°C and the temperature is precisely controlled (±0.1°C). We installed another incubator with a temperature set to 4°C (4.2 ± 0.4°C); the incubator at 4°C is a new feature of PXS2 for a better success rate in crystallization. The 4°C incubator has room for 400 plates and an additional RockImager2. After setting up the crystallization drops at 20°C, the plates can be immediately moved into the 4°C incubator. Owing to the dedicated imager at 4°C, scheduled observations can be performed without changing the temperature of the crystallization plate.

A previous report showed that the incubation temperature has a profound effect (Ng *et al.*, 2016 ▸). We tested the influence of temperature on the crystallization process using our incubators at 20 and 4°C. Four reference protein samples, lysozyme (Wako Pure Chemical, Japan), thaumatin (Wako Pure Chemical, Japan), thermolysin (Nacalai Tesque, Japan) and glucose isomerase (Hampton Research, California, USA), were crystallized under 768 different conditions at 4 and 20°C by PXS2. Remarkable differences were found between the two temperatures (Table 2 ▸), suggesting that crystallization at not only 20°C but also 4°C increases the success rate. Indeed, a follow-up survey of our in-house projects suggested that crystallization at both 20°C and at 4°C was effective. Of the 21 cases of successful crystallization screening, four cases gave crystals only at 4°C. Therefore, it is better to try both temperatures for crystallization screening if there is a sufficient amount of sample.

### 2.3. New functions for membrane-protein crystallization   

#### 2.3.1. Overview and test-sample preparation   

The original PXS used the vapor-diffusion method, which is mainly suitable for the crystallization of soluble proteins. Along with the increased demand for crystal structures of membrane proteins, our users have increasingly requested high-speed and large-scale crystallization screening for membrane proteins. Among several available membrane-protein crystallization methods (Ishchenko *et al.*, 2017 ▸), we selected two crystallization methods, the bicelle and LCP methods, for PXS2.

To confirm the performance of PXS2 with membrane proteins, we used two test samples, *Neisseria meningitidis* PorB as a β-barrel protein (Tanabe *et al.*, 2010 ▸) and *Rubrobacter xylanophilus* rhodopsin (RxR) as an α-helical protein (Hayashi *et al.*, 2020 ▸). Concentrated PorB (∼15 mg ml^−1^) and RxR (∼11 mg ml^−1^) were used to test the improved system. For the bicelle method, PorB or RxR was mixed with bicelle solution (as described below). For the LCP method, PorB or RxR was mixed manually with monoolein (Nu-Chek Prep, Minnesota, USA) in a protein:lipid ratio of 2:3(*w*:*w*). The mixed sample was dispensed on a film sandwich in a 60 nl drop and was overlaid with 800 nl precipitant solution using the mosquito LCP.

#### 2.3.2. Bicelle method   

**Figure 5:**
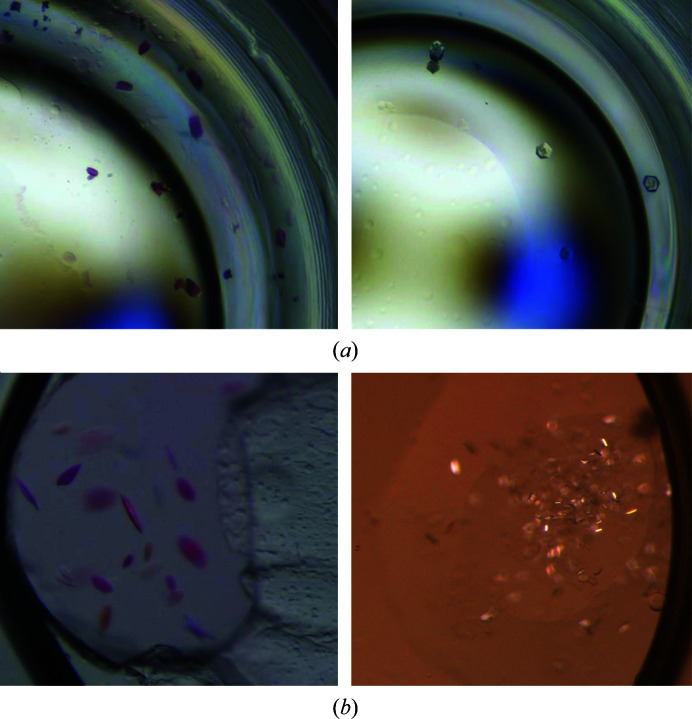
Crystals of membrane-protein samples. (*a*) Crystals obtained by the bicelle method. RxR (left) and PorB (right) crystals were grown under conditions consisting of 0.05 *M* HEPES pH 7.5, 2.5 *M* ammonium sulfate and of 35%(*v*/*v*) MPD, 0.1 *M* Tris pH 8.5, 0.2 *M* ammonium sulfate, respectively. The premixed bicelle solutions were purchased from Molecular Dimensions (Sheffield, UK). (*b*) Crystals obtained by the LCP method. RxR (left) and PorB (right) crystals were grown under conditions consisting of 0.1 *M* HEPES pH 7.5, 1 *M* sodium sulfate, 0.05 *M* cadmium sulfate and of 20%(*v*/*v*) PEG 300, 10%(*w*/*v*) glycerol, 0.1 *M* imidazole, 1 *M* ammonium sulfate, respectively.

Bicelles, which are typically composed of a mixture of long-chain phospholipids, short-chain fatty acids and/or detergent, are lipid bilayer membranes with a disk-like shape. As an initial crystallization condition, the purified target membrane protein is mixed with bicelle solution in a 4:1(*v*:*v*) (protein:bicelle) ratio. The concentration of the bicelle solution was 25–40% 2.8:1(*v*:*v*) 1,2-dimyristoyl-*sn*-glycero-3-phosphocholine (DMPC):3-[(3-cholamidopropyl)dimethylammonio]-2-hydroxy-1-propanesulfonate (CHAPSO). The protein/bicelle mixture and KEK plates were incubated for 30 min on ice prior to setting up the sample in PXS2. Since the protein/bicelle mixture tends to be gelatinous at room temperature and to be difficult to dispense properly, the crystallization setup is performed every two crystallization plates. The aspirate/dispense protocol of the mosquito LCP, which is used for the crystallization, is half the speed of the usual vapor-diffusion protocol. All other procedures are the same as in the vapor-diffusion method. The results of the bicelle crystallization of test samples are shown in Fig. 5 ▸(*a*).

#### 2.3.3. LCP method   

**Figure 6:**
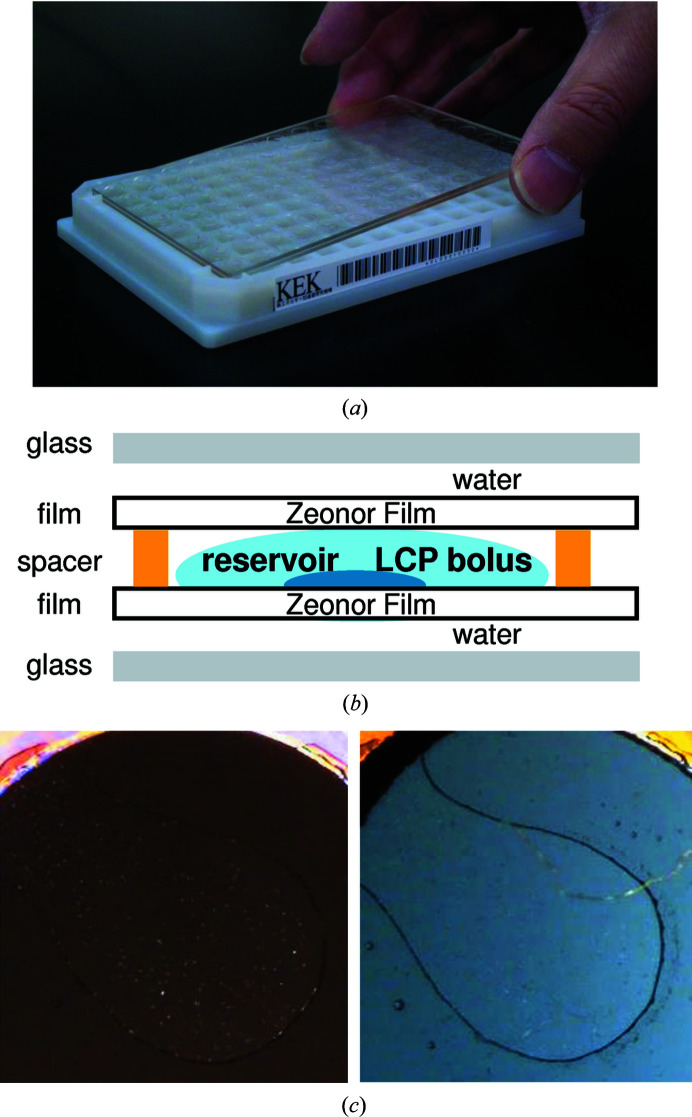
KEK-style membrane sandwich LCP crystallization. (*a*) A KEK original adaptor (bottom) is in the SBS format and can be used with PXS2 because the adaptor is compatible with the KEK crystallization plate. A film sandwich LCP plate supported by glass plates (top) fits onto the adaptor. (*b*) Schematic drawing of a cross section of the KEK-style LCP plate. (*c*) Comparison of the crystallization-drop images under a polarized condition between glass-supported (KEK-style, left) and plastic-supported (MD Diffrax, right) LCP plates.

In the LCP method, the following experimental procedures are needed to set up the crystallization drops. Initially, a protein sample is mixed with a lipid solution in connected airtight syringes. Next, the mixture is dispensed to prepare LCP drops by an automated or a manual dispenser. The dispensed drops must then be covered with glass or other materials. Since these procedures are hard to automate, we decided to prepare the LCP crystallization drops manually. The setting up of the LCP crystallization drops is performed using the mosquito LCP, and only the storage and scheduled observation of the LCP plate are performed using PXS2. Since the size of the LCP plate differs from that of the KEK plate, we made an original adaptor with the same shape and size as the KEK plate (Fig. 6 ▸ *a*). A barcode was affixed to the side of the adaptor to manage the plate.

While glass sandwich plates have frequently been utilized in the LCP method, the top glass must be broken when the crystals are harvested for X-ray diffraction experiments (Li *et al.*, 2012 ▸). This procedure, however, is very difficult, especially when crystals appear in the sponge phases. In addition, the crystals are sometimes lost during crystal harvesting. Therefore, techniques using a film instead of a glass have been developed (Huang *et al.*, 2015 ▸; Axford *et al.*, 2016 ▸). Crystallization-plate kits for the film sandwich technique are commercially available (Table 3 ▸). In these kits, a glass or plastic support is required to prevent evaporation of the LCP mixture solution. We combined the advantages of the glass sandwich and film sandwich techniques. PXS2 utilizes the film sandwich as the basic format, but also uses glass for the outer support material (Fig. 6 ▸ *b*). The inner film was purchased from Molecular Dimensions, UK (catalog No. MD11-82). The outer glass (109 × 73 × 1 mm) is custom-made by Matsunami Glass, Japan and can be used repeatedly. The inner film is covered by another thin film to prevent dust when it is supplied. For easy handling, dispensing of the LCP drops to the film by the mosquito LCP is performed without peeling off the thin film to prevent unnecessary adhesion. After this, the thin cover film is peeled off and the top cover glass is placed.

After the LCP crystallization setup using the mosquito LCP, the transport rail robot brings the plate into an incubator. Since the lipid cubic phase is optically isotropic, it is advantageous to use a crossed polarizer to detect submicrometre-sized birefringent protein crystals (Cherezov & Caffrey, 2003 ▸). It is sometimes necessary to rotate the polarizer according to the crystal direction. In our system, users can observe LCP drops using the RockImager2 under three different polarized conditions; *i.e.* with the angles of the polarizer set to 0°, 45° and 85°. The captured images are observed in the same way as the usual KEK plates. This method enables users to find crystals in the LCP drop easily. It is of note that the glass support is better than the plastic support when observing crystals with a polarizer. The KEK-style LCP format of a film sandwich with a glass support provided good observation of the crystallization drops (Fig. 6 ▸ *c*). Results for the test membrane-protein samples with LCP crystallization are shown in Fig. 5 ▸(*b*).

## 3. Conclusions and perspectives   

The original PXS operated for more than 14 years and played an important role not only in a structural genomics project (Hiraki *et al.*, 2006 ▸) but also in structural biology research (Katsuyama *et al.*, 2018 ▸; Koentjoro *et al.*, 2018 ▸; Nakashima *et al.*, 2018 ▸), including structure-based drug discovery. In this study, we upgraded PXS to PXS2 in order to respond to the demands of users and meet the requirements for advanced structural studies. The upgrade was performed by replacing modules of PXS with new modules. The main improvements are a reduction in the amount of sample that is required for crystallization screening and the addition of new options for membrane-protein crystallization. These improvements realize high-throughput crystallization screening for wider protein targets in a labor-saving manner, reducing the bottleneck of MX. The original PXS set up 676 plates per year on average (from 2003 to 2016) and PXS2 set up 894 plates per year (from 2017 to mid-2020), including membrane proteins. The outcome of PXS2 is beginning to become apparent (Hayashi *et al.*, 2020 ▸; Koiwai *et al.*, 2020 ▸; Kuwabara *et al.*, 2020 ▸). Our PXS2 system can be accessed through the BINDS (Basis for Supporting Innovative Drug Discovery and Life Science Research) project, which is a Japanese national research project to establish an innovative platform for expediting the therapeutic applications of early-stage drug-discovery and medical technology advances. One technician handles the setup schedule of PXS2 and operates the setup for external samples. Samples inside the Structural Biology Center are set up by the technician or a researcher in the center. Since it only takes about half an hour to set up eight plates, anyone who wants to use PXS2 can use it at any time. Sometimes there is a request to set up a large number of plates, and even then these can be set up the next day.

We are working on two developments to improve the efficiency of PXS2. Firstly, we are developing a system for the automatic evaluation of acquired images of the crystallization drops. While the acquired images are automatically stored and can be accessed through the internet, these images must be checked by researchers manually. Since manually checking all images is a time-consuming process, we are developing automated scoring software using machine learning (Liu *et al.*, 2008 ▸; Bruno *et al.*, 2018 ▸; Miura *et al.*, 2018 ▸). Integration of the automated scoring software into PXS-PReMo will enable researchers to reduce the time and effort spent on finding crystals in a number of acquired images. The second development in efficiency will be achieved through *in situ* X-ray diffraction data collection (Bingel-Erlenmeyer *et al.*, 2011 ▸). At the PF beamlines, it is already possible to directly irradiate a crystal in a crystallization plate with X-rays without fishing it out (Yamada *et al.*, 2016 ▸). Cryo-treatment may influence native macromolecular structures in cryo-MX methods, but measurements in the unfrozen state, such as *in situ* data collection, will avoid such artifacts. One of the goals of our efficiency improvements is to integrate automated crystal detection and automated *in situ* data collection. When the automatic drop scoring and *in situ* X-ray diffraction data collection are combined through the collaboration of PXS-PReMo and PReMo, it will be possible to realize faster X-ray crystallographic structure determination.

## Figures and Tables

**Table 1:** Specifications of the original and the improved systems

	Original PXS	Improved PXS2
Crystallization methods	Vapor diffusion	Vapor diffusion, bicelle, LCP
Dispensed sample volume (µl)	0.5	0.1–0.2
Plate-making speed† (one 96-well plate)	36 s	3 min 30 s (vapor diffusion), 3 min 50 s (bicelle)
Scale of one batch (vapor diffusion)	80 plates, 10 screen kits, 8 protein samples	8 plates, 8 screen kits, 1 protein sample
Plate-sealing method†	Adhesive	Crimping
Plate incubator (20°C)	1100 plates (4 units)	1530 plates (5 units)
Plate incubator (4°C)	—	400 plates (1 unit)
Observation resolution	0.3 million pixels	5 million pixels
Observation speed (one 96-well plate)	1 min 20 s	4 min 50 s
SONICC observation module	—	SHG, UV-TPEF

† The descriptions of the plate-making speed and plate-sealing method apply to the vapor-diffusion and bicelle methods but not the LCP method.

**Table 2:** Numbers of conditions under which crystals appeared Among the 768 conditions tested, the numbers of conditions under which crystals appeared were counted five days after crystallization. The crystallization kits used were Crystal Screen, Crystal Screen 2, PEG/Ion 1, PEG/Ion 2, Index and MembFac from Hampton Research, California, USA, Wizard Classic 1 and 2 and Wizard Cryo 1 and 2 from Rigaku, Japan, The Protein Complex and PEGs II Suites from Qiagen, Germany and Stura Footprint from Molecular Dimensions, UK.

	20°C	4°C
Lysozyme (50 mg ml^−1^)	67	195
Thaumatin (20 mg ml^−1^)	11	6
Thermolysin (15 mg ml^−1^)	12	17
Glucose isomerase (20 mg ml^−1^)	85	160

**Table 3:** Summary of LCP plates

		Film sandwich		
	Glass sandwich	MD, Diffrax†	MiTeGen, IMISX†	KEK
Drop observation	Good	Not good	Good	Good
X-ray experiments				
Picked-up crystals	Pickup difficult	—	—	—
Cut film	—	Good	Good	Good
Plate (*in situ*)	Impossible	Possible	Possible	Possible
Film material	—	Zeonor	COC	Zeonor
Support material	—	Plastic	Glass	Glass
Plate format compatible with PXS2	No	No	No	Compatible

† The Diffrax and IMISX plates were supplied by Molecular Dimensions (MD), UK and MiTeGen, New York, USA, respectively.
